# Unusual Synchronous Lung Tumors: Mucoepidermoid Carcinoma and Mucinous Adenocarcinoma

**DOI:** 10.1155/2014/183617

**Published:** 2014-02-18

**Authors:** Ana M. Ponea, Creticus P. Marak, Ying Sun, Achuta Kumar Guddati, Amit S. Tibb

**Affiliations:** ^1^Division of Pulmonary and Critical Care Medicine, Montefiore Hospital, Albert Einstein College of Medicine, Yeshiva University, New York, NY 10467, USA; ^2^Division of Pulmonary Medicine, Department of Medicine, Tahlequah City Hospital, Tahlequah, OK 74464, USA; ^3^Department of Pathology, Montefiore Hospital, Albert Einstein College of Medicine, Yeshiva University, New York, NY 10467, USA; ^4^Department of Internal Medicine, Massachusetts General Hospital, Harvard Medical School, Harvard University, 50 Fruit Street, Boston, MA 02114, USA

## Abstract

Primary mucoepidermoid tumors of the lung are rare entities. Synchronous primary malignancies of the lung involving mucoepidermoid carcinoma and mucinous adenocarcinoma are even rarer and constitute a unique set of patient population. The presentation, diagnosis and treatment strategies for this patient population are not well described. In most cases, the diagnosis of synchronous primary lung malignancy is made after pathological examination of the resected lung specimen. Molecular and genetic analysis is now being used to supplement the diagnosis of synchronous primary lung malignancies. In this work, we briefly discuss the current state of knowledge of this unique combination of primary lung malignancies and describe the clinical presentation and management of a patient with a rare combination of synchronous primary lung malignancies.

## 1. Introduction

Synchronous tumors are defined as two or more primary neoplasms which are detected simultaneously. They differ from metachronous tumors which are detected after an intervening interval in their epidemiology, prognosis, and management [1]. Precancerous lesions have been found at a higher frequency in patients with multiple primary lung cancers, but the genetic basis of such malignancies is yet to be elucidated [2, 3]. It is possible that the development of synchronous tumors is related more to environmental exposure than to genetic predisposition as seen by the higher incidence of synchronous tumors in workers exposed to chromate [4]. However, there is some evidence to show that genetic predisposition may play a role as multiple primary lung cancers have been observed to be inherited in some families [5]. Mutations in the p53 protein and allelic loss of heterozygosity have been shown to be associated with synchronous lung primary malignancies [6, 7]. Similar trend with malignancies involving other organ systems has been noted in patients with significant smoking history providing credence to the hypothesis of field cancerization [8].

The diagnosis of true synchronous primary lung tumors with different histologies has been difficult due to two reasons: some patients with multiple primary lung tumors with different histologies have been found to have identical genetic changes suggesting a monoclonal origin and some with patients with identical histologies have been found to have different clonal origins [9–11]. This is complicated by the fact that regional heterogeneity in tumor grade can be observed in individual neoplasms and metastases may have different tumor grades when compared to their primary lesions [12]. Notably, imaging with fludeoxyglucose-positron emission tomography (FDG-PET) has been noted to have differences in uptake between different lesions in patients with synchronous primary lung tumors [13]. However, the clinical significance of this finding is not yet clear and has not factored into treatment strategies. It is also possible that the second primary malignancy may represent a metastasis from an extrapulmonary site, and hence a thorough evaluation for other primaries is warranted [14]. Recent studies have shown microsatellite analysis to be useful in differentiating the multiple primary tumors from each other and also from metastases [15].

Mucoepidermoid carcinoma is a common salivary gland tumor which is often seen in the digestive system and the respiratory tract. They were first described in 1945 by Stewart et al. [16] in the salivary glands and in the tracheobronchial tree by Smetana et al. [17]. Metastasis to the skeletal muscle, pericardium, pleura, mediastinum, central nervous system, gastrointestinal tract, and kidney has been described [18–21]. Primary pulmonary mucoepidermoid carcinomas are rare and comprise 0.1-0.2% of primary lung malignancies [22]. Synchronous mucoepidermoid and mucinous adenocarcinomas are even rarer and there are very few reports in medical literature which describe this combined entity.

## 2. Case Description

**Figure 1:**
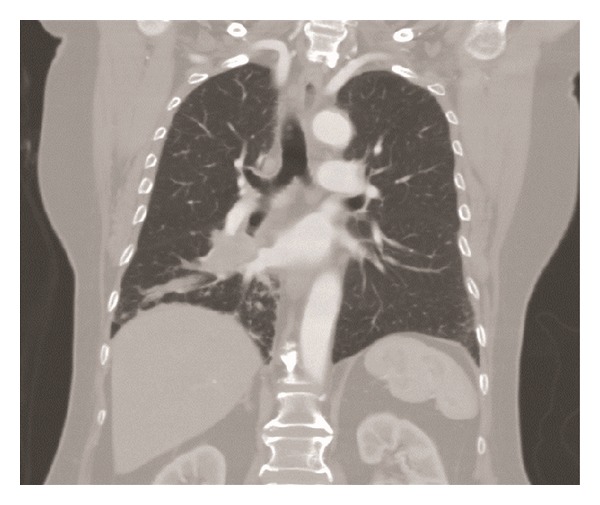
Transverse section on a chest CT demonstrating the presence of tumor lesions in the right middle and lower lobes.

**Figure 2:**
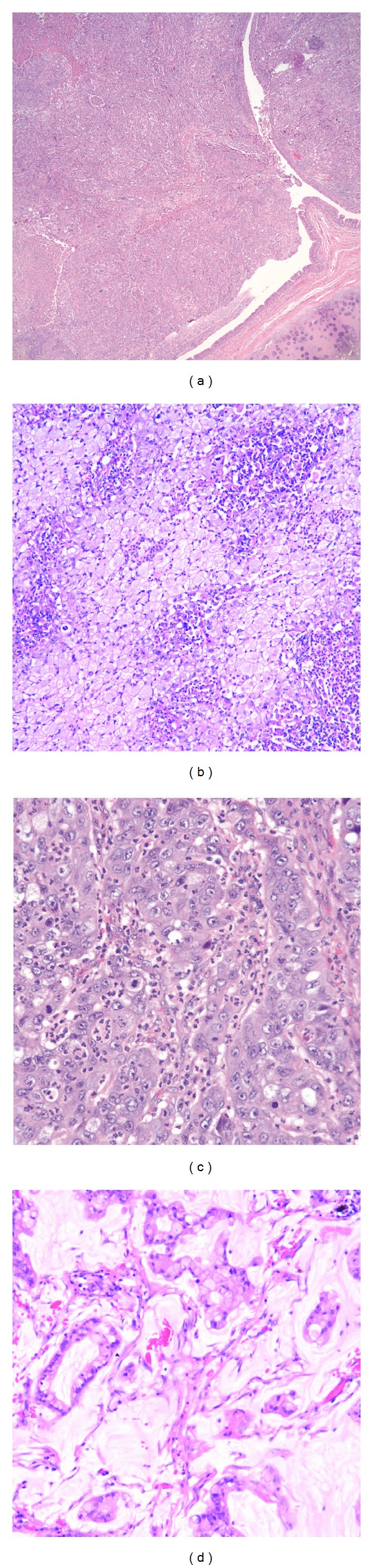
(a) Hematoxylin and eosin (H & E) stain showing endobronchial invasion of mucoepidermoid carcinoma. (b) H & E staining showing the mucinous component of mucoepidermoid carcinoma. (c) H & E staining showing the solid component of mucoepidermoid carcinoma. (d) H & E staining of mucinous adenocarcinoma.

**Figure 3:**
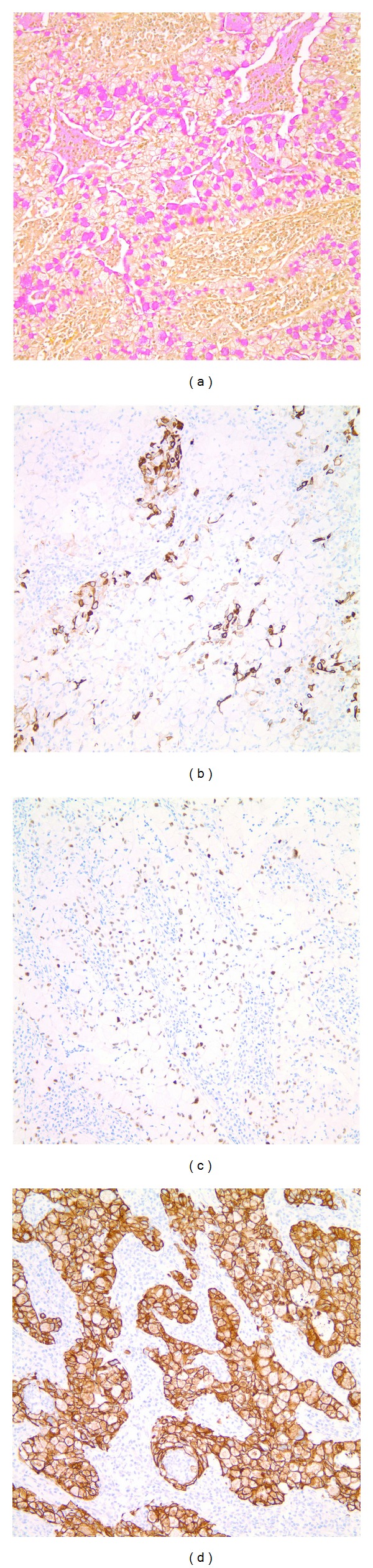
Immunohistochemistry showing staining with mucicarmine (a), CK 5, and 6 (b), p63 (c) and CK 7 (d).

The patient is a 68-year-old lady with a past medical history significant for hypertension, hyperlipidemia, and hypothyroidism who presented with complaints of lower abdominal pain, diarrhea, and bright red blood per rectum. She was a retired medical assistant who had quit smoking 3-4 weeks prior to presentation after having smoked half pack a day for 30 years. Her home medications included lisinopril, simvastatin, aspirin, levothyroxine, and calcium and vitamin D supplementation. Her review of systems was positive for mild intermittent dry cough for the past 1.5 months. She denied fever, chills, weight loss, productive cough, hemoptysis, night sweats, and changes in urinary habits. She had unlimited exercise tolerance. Her physical examination in the emergency department was unremarkable except for mild tenderness to palpation over the lower abdomen. A computed tomography (CT) scan of the abdomen showed sigmoid and distal ascending colon colitis and incidentally identified a right hilar mass. The mass was in the superior segment of the right lower lobe and extended throughout the lower lobe along the pulmonary vessels (Figure 1). The central part of the mass was enhancing with hypodensity noted peripherally suggestive of necrosis. The dominant portion of the mass measured up to 6 cm transverse × 3.4 cm antero-posterior. The tracheobronchial tree was found to be patent. An additional irregular, pleural based mass in the superior segment of the right lower lobe was also identified which measured 3.9 cm transverse × 1.5 cm anteroposterior. An additional pleural nodularity was noted in the inferior portion of the right lower lobe. These findings were highly suggestive of a right lower lobe malignancy. There was no significant mediastinal, axillary, or hilar adenopathy noted except for a subcarinal (9 mm in short axis) and paratracheal lymph node (5 mm in short axis). She underwent bronchoscopy which showed a friable exophytic endobronchial mass in the right lower lobe at 1 cm from the bronchus intermedius, rest of the anatomy normal. Bronchial brushings and endobronchial biopsies × 3 were done. Immunostaining was positive for mucicarmine, cytokeratin 7 (CK7), p63, focally positive for cytokeratins 5,6 (CK 5,6), negative for synaptophysin, thyroid transcription factor-1 (TTF-1), epidermal growth factor receptor (EGFR), anaplastic lymphoma kinase (ALK) and Napsin A (hematoxylin and eosin stains are shown in Figures 2(a), 2(b), 2(c) and 2(d); immunostaining in Figures 3(a), 3(b), 3(c), and 3(d)). Positron emission tomography-CT (PET-CT) showed a large focus of intense uptake in the lower right lung medially, corresponding to a hilar mass extending to the lower lobe seen on chest CT. No other focal abnormalities are noted. She underwent right sided Video-Assisted Thoracic Surgery (VATS) with right middle and lower lobe bilobectomy with lymph node dissection. Pathology showed 2 separate synchronous carcinomas (1st tumor: nonsmall cell carcinoma, favor mucoepidermoid; 2nd tumor: mucinous adenocarcinoma). It was deemed that the patient had 2 distinct primaries, both staged at T2N1. Patient refused chemotherapy and has been doing well after procedure.

## 3. Discussion

Patients with synchronous lung tumors have been shown to have a worse prognosis when compared with patients with lung malignancy at a similar stage [23, 24]. In the past, pneumonectomy was associated with poor outcomes in patients with multiple synchronous primary tumors, but this trend has changed recently with aggressive surgical treatment [25–27]. This change has been attributed to a relative increase in patient population with tumors at an earlier stage, possibly linked to better detection techniques and increased surveillance. While tumor size and lung function have been shown to be independent predictors of survival, female gender seems to be prognosticator for better outcomes [1]. Patients with metachronous tumors on the other hand have been shown to have a better survival rate when compared to patients with synchronous lung primary malignancies [28, 29].

More than 50% of mucoepidermoid carcinomas have a characteristic translocation between chromosomes 11q and 19p [30, 31]. This rearrangement results in the formation of a fusion protein between mucoepidermoid carcinoma translocated-1 (MECT1) and mastermind-like protein 2 (MAML2) genes [32]. MECT1 protein activates the c-AMP response element-binding mediated transcription and MAML-2 protein is a part of notch signaling [33, 34]. The formation of this fusion protein has been shown to be pathognomic for most of mucoepidermoid carcinomas [34, 35]. It was initially thought that presence of the fusion protein was associated with better median survival, but recently it has been shown that these patients did not have significant disease-free survival despite better disease-specific survival [30, 36]. The role of the fusion protein as a prognosticator remains controversial [37, 38].

Surgical resection has been the mainstay of treatment for low grade mucoepidermoid carcinomas but neodymium yttrium aluminum garnet (Nd-YAG) laser has also been used [39, 40]. Adjuvant chemotherapy has been reserved for unresectable and advanced cases with limited success. The mortality of high grade mucoepidermoid carcinomas continues to remain high [41]. In rare cases where the tumor is limited to the same lobe, surgical resection has been curative for synchronous lesions involving both mucoepidermoid and adenocarcinoma [42].

## 4. Conclusion

The patient described here underwent surgery with an excellent outcome. This case report serves to illustrate the rarity of synchronous lung malignancies involving mucoepidermoid carcinoma and mucinous adenocarcinoma. It also demonstrates that early surgical intervention in patients with synchronous lung malignancies at early stages may have favorable outcomes despite the involvement of more than one lung lobe.
